# Metaviromics of Namib Desert Salt Pans: A Novel Lineage of Haloarchaeal Salterproviruses and a Rich Source of ssDNA Viruses

**DOI:** 10.3390/v8010014

**Published:** 2016-01-08

**Authors:** Evelien M. Adriaenssens, Leonardo Joaquim van Zyl, Don A. Cowan, Marla I. Trindade

**Affiliations:** 1 Centre for Microbial Ecology and Genomics, Genomics Research Institute, University of Pretoria, Natural Sciences II, Lynnwood Road, 0002 Pretoria, South Africa; don.cowan@up.ac.za; 2 Institute for Microbial Biotechnology and Metagenomics, University of the Western Cape, 7535 Bellville, Cape Town, South Africa; vanzyllj@gmail.com (L.J.V.Z.); ituffin@uwc.ac.za (M.I.T.)

**Keywords:** Namib Desert playa, metaviromics, environmental viromics, hypersaline, haloarchaeal viruses, ssDNA viruses, *Gokushovirinae*, *Microviridae*

## Abstract

Viral communities of two different salt pans located in the Namib Desert, Hosabes and Eisfeld, were investigated using a combination of multiple displacement amplification of metaviromic DNA and deep sequencing, and provided comprehensive sequence data on both ssDNA and dsDNA viral community structures. Read and contig annotations through online pipelines showed that the salt pans harbored largely unknown viral communities. Through network analysis, we were able to assign a large portion of the unknown reads to a diverse group of ssDNA viruses. Contigs belonging to the subfamily *Gokushovirinae* were common in both environmental datasets. Analysis of haloarchaeal virus contigs revealed the presence of three contigs distantly related with His1, indicating a possible new lineage of salterproviruses in the Hosabes playa. Based on viral richness and read mapping analyses, the salt pan metaviromes were novel and most closely related to each other while showing a low degree of overlap with other environmental viromes.

## 1. Introduction

Research into the viral community structure of saline environments has focused mainly on marine environments with a limited number of studies looking at moderate or hypersaline lakes and solar salterns [1,2,3,4,5,6,7]. The study of extremophilic phages has revealed new mechanisms for host lysis, led to the introduction of new viral families, demonstrated interactions between phage and host proteins which are unlike those normally observed for mesophilic phages, shown phages with leaderless promoters, and has been a source of novel enzymes [8,9,10,11,12,13]. Investigating viruses infecting extremophiles will therefore shed light on the roles of their respective hosts in nature, as well as on the phage–host relationships as well as being a source of new tools for molecular biology research [14,15,16].

Isolation studies of hypersaline environments have revealed four main types of virus morphologies; spindle shaped or fusiform, head-tail, icosahedral and pleomorphic, while filamentous and rod-shaped viruses have also been observed by transmission electron microscopy (TEM) [2,17,18,19]. Not all of these morphologies are necessarily seen in any hypersaline environment, as shown by the viral diversity of Mono Lake (CA, USA) where only tailed and icosahedral viruses were found [20]. Currently, there are only a limited number of haloarchaeal virus genomes deposited in public databases, mainly from head-tail viruses, but also of pleomorphic viruses which can have single or double-stranded DNA genomes [21,22,23,24]. Shotgun metaviromics has been used as an alternative method for obtaining genome information for diversity studies in the absence of a universal viral marker gene. This also eliminates the bias created by viral isolation studies which rely on culturing of the host bacterium. For hypersaline crystallizer ponds, no viruses have been isolated infecting the two main archaeal components, *Haloquadratum walsbyi* and *Salinibacter ruber* [4]. Using culture-independent fosmid library cloning of metaviromic DNA, a potential phage genome for *H. walsbyi* was identified and named EHP1, but host assignment was very speculative [25]. Fosmid-based viral genomes have been assigned to a putative host with a higher reliability using cluster analysis based on tetranucleotide and codon usage binning, and confirmed with CRISPR (Clustered Regularly Interspaced Short Palindromic Repeats) spacer identification, revealing, among others, viruses of *H. walsbyi* [26]. The diversity of viruses and phages in the Namibian desert has not been adequately explored with only a handful of studies which describe phage isolation [27], limited sequencing [28] and one metagenomic study, focused on a niche environment, that of hypoliths [29]. Thus, much work still has to be done in characterizing the diversity of bacteriophages and viruses from this extreme environment.

In this study, we have sequenced metaviromes of two different salt pans (playas) in the Namib Desert, namely the Hosabes playa and another near the town of Swakopmund (Eisfeld). Playas found in the Namib Desert are moist, salt-covered, sediment-filled depressions which form in drainage channels whose surface and groundwater flow is obstructed by linear bedrock outcrops, in particular dolerite dykes, and their mineralogy and geochemistry has been well-studied by others [30,31,32,33,34]. They have a reported salinity of 3%–15% depending on the distance from the source, the depth of the pool and the time of day (evaporation), featuring halite (NaCl) and gypsum crusts (CaSO_4_·2H_2_O), with the Hosabes playa also containing the rare nitrate mineral Humberstonite (K_3_Na_7_Mg_2_(SO_4_)_6_(NO_3_)_2_·H_2_O) [30,31,32,33]. Follow-up studies indicated that the Hosabes playa is perennial and is not chemically different from the coastal Eisfeld playa which contains no marine water signals [34]. Interestingly, the Hosabes playa has been reported to reach temperatures as high as 50 °C in the water column [31]. While the temperature range does not qualify these sites as extreme, the salinity range makes them a potential home to both halotolerant and halophilic microorganisms [35], and the combinations of the isolated and unique location, higher temperatures and salinities found here could indicate that they also harbor unique viral populations.

The Hosabes and Eisfeld playas were identified as sites of interest for bioprospecting for novel enzymes, because of their salt content and their unexplored nature. The use of metaviromics for the exploration of new enzymes was suggested previously, taking into account the high density of coding sequences in viral genomes and the absence of a cultivation step [36]. This way, using a screening approach, a novel thermostable DNA polymerase was identified from a Yellowstone hot spring metavirome [11,37]. In this paper, we aimed to describe the diversity of viruses in the chosen salt pans, tried to assign the unidentified fractions of the metaviromes and investigate the uniqueness of the viral communities in these sites.

## 2. Materials and Methods

### 2.1. Sample Collection and Processing

**Figure 1:**
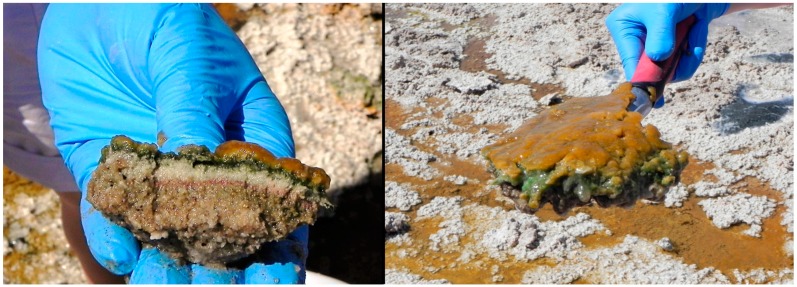
Eisfeld playa (Swakopmund Saline) microbial mat as collected in this sample and mixed with water from the spring (Picture courtesy of E. Rybicki).

The two sample sites were located in the Namib Desert approximately 124 km apart, one near the Gobabeb Training and Research Station (Hosabes: Gobabeb Saline or GS) at 23°30′25.75′′ S, 15°4′16.50′′ E, about 100 km inland from the town of Walvis Bay, while the Eisfeld site is located 21.5 km North East of Swakopmund (Swakopmund Saline or SS) at 22°29′5.02′′ S, 14°34′18.06′′ E [30]. From each site, 50 L of water, including any microbial mat formations which are depicted in Figure 1, was collected in sterile drums. Inclusion of the microbial mats was to insure representation of the most diverse viral community present in the salt springs. The water was collected from the flowing streams after digging a depression into the spring bed, letting it fill with water and then scooping it out into 2 × 25 L drums. The microbial mats from this depression were broken into pieces and forced through the drum opening, thus making a mix including microbial mat formations and water from the salt pan spring. The water was processed on site at ambient temperature; it was first filtered through 1 μm (CR0101006) and 0.22 μm (KVGLA10HH1) nitrocellulose filters (Millipore, Merck, Darmstadt, Germany) and subsequently concentrated with tangential flow filtration (TFF) using a prep-scale 30 kDa cut-off cartridge (Millipore; CDUF006LT) to a volume of about 100 mL. The system was sterilized with 0.1 M NaOH and rinsed with distilled water.

Further sample processing occurred at the Institute for Microbial Biotechnology and Metagenomics (IMBM) at the University of the Western Cape (South Africa), where the concentrates were stored for two weeks at 4 °C until processing.

On site, metadata was collected (from 2010 to 2015). The pH was measured with narrow range strips (Merck, Darmstadt, Germany). The temperature and conductivity were measured using probes (Hanna Instruments, HI 9040 microcomputer thermometer, HI 8633 conductivity meter) which were either submersed in the stream or in water collected with a syringe from the stream and measured in a falcon tube. The conductivity meter was calibrated at 25 °C and 12.88 mS/cm buffer solution. Total dissolved solids and total salinity were calculated based on the conductivity at 25 °C.

### 2.2. Library Construction and Sequencing

Two independent DNA extractions were performed per site (GS and SS), using 25 mL of TFF (tangential flow filtration) sample per extraction, to limit any bias arising from the DNA extraction protocol. To ensure that DNAse and RNAse treatments performed later in the protocol were successful, the sample was first dialyzed against 5 L of 100 mM Tris buffer (pH8.0) using Snakeskin™ dialysis tubing (Life Technologies, Thermo Fischer Scientific, Waltham, MA, USA; 10 kDa cutoff) to remove excess salt. The suspension was concentrated down to the original 25 mL by dialysis against a 10% *w*/*v* solution of polyethylene glycol (PEG) 10,000. The dialyzed samples were inspected using fluorescence microscopy and no noticeable decrease in viral particles was observed. Virus particles were collected by centrifugation in a Beckman JA20 rotor at 43,667× *g* for 6 h in autoclaved 30-mL Nalgene polypropylene copolymer (PPCO) tubes, and the individual pellets were resuspended in 1 mL Tris buffer. The suspension was treated with DNase I (catalog no. EN0521; Fermentas) and RNase A (catalog no. EN0531; Fermentas) at a final concentration of 0.1 μg/mL at 37 °C for 2 h. The efficacy of DNAse treatment was assessed in two ways. The first was to remove an aliquot of the sample to which DNAse had been added and combining it with bacterial genomic DNA which was incubated at 37 °C alongside the sample. This digest was analyzed by agarose gel to determine if the bacterial genomic DNA was degraded. A portion of the DNAse treated sample was then used as template for PCR (35 cycles) to amplify bacterial 16S rRNA genes, including controls spiked with bacterial genomic DNA to check for PCR inhibitors from the sample. If no product was detected when loading the entire PCR reaction (50 μL) on an agarose gel, we proceeded with DNA extraction. Proteinase K (Fermentas) was added to a final concentration of 1 μg/mL and the suspension incubated at 55 °C for 2 h. SDS was added to a final concentration of 0.6% (*w*/*v*) and incubated at 37 °C for 1 h. Nucleic acids were purified by performing two rounds of phenol-chloroform-isoamyl alcohol (25:24:1) phase separation followed by one round of chloroform-isoamyl alcohol (24:1) phase separation. DNA was precipitated by the addition of 1/10 volume of sodium acetate (3 M; pH 5.2) and 2 volumes of 100% ethanol at 4 °C overnight. Samples were centrifuged at 29,000× *g* for 10 min to pellet the DNA, which was resuspended in 30 μL of TE (Tris-EDTA) buffer. The two separate DNA extractions per site were pooled for the whole genome amplification. Ten nanograms of DNA from each sample was used to perform Phi29 amplification (GenomiPhi HY DNA amplification kit; catalog no. 25-6600-20; GE Healthcare, Little Chalfont, UK) using the manufacturer’s recommendations. The DNA was purified using the Qiagen gel extraction kit (Qiaex II; catalog no. 20021; Qiagen, Hilden, Germany) for use in preparation of sequencing libraries. Library preparation included a 10% phiX V3 spike as per the manufacturer’s instructions (Illumina Nextera XT guide) with the Illumina Nextera XT library prep kit/MiSeq reagent kit V2 (Illumina, San Diego, CA, USA). Three libraries were constructed for DNA from each site, to capture a wider variety of DNA species for sequencing. The amplified DNA was sequenced 2 × (forward and reverse sequencing) 250-bp reads, on the Illumina MiSeq platform located at the University of the Western Cape, Cape Town, South Africa.

### 2.3. Raw Read Processing and Assembly

The quality of the raw read files was first checked with FastQC [38] getting a pass for the forward reads (R1 files) and a fail or a warning for the reverse reads (R2 files). Using in-house scripts the reads were then filtered for a minimum quality of 25 and trimmed at the 3′ end in a sliding window until read quality was at least 25 with a minimum length of 150 bp. This yielded 19,754,486 reads with an average length of 174 bases for GS and 20,662,380 reads with an average length of 172 bases for SS. The reads mapping to phiX version 3 as supplied by Illumina were removed using bowtie2 at the default settings (version 2.2.3). Of the post-quality control (QC) forward read files, random subsets were taken for submission to MetaVir [39,40] which has a limit of 2.5 million reads per metavirome.

The post-QC reads were assembled using CLC Genomics Workbench as paired files (3 × 2 read files per site), using the default settings, and any residual contigs showing more than 99% sequence identity with phiX were removed. Additional contamination screens were performed using the whole genome shotgun (WGS) submission pipeline of the National Center for Biotechnology Information (NCBI) and any contigs showing contamination were removed. For GS, the assembly resulted in 11,304 contigs with a minimum length of 200 bases at an N50 of 530 and a maximum of 74,962 bases. The assembly of the SS reads gave 22,352 contigs of minimum 200 bases at an N50 of 457 and a maximum contig size of 23,854 bases. These contig files were also submitted to MetaVir and VIROME for analysis [39,40,41]. The contig datasets are available from GenBank under BioProject PRJNA287316, accession numbers LFUF00000000 and LFUG00000000. The versions described in this paper are LFUF01000000 and LFUG01000000.

### 2.4. *In Silico* Analyses

Contig networks for the Namib Saline sites were generated by first mapping the post-QC reads to their respective contigs using bowtie2 [42]. The reads were mapped as paired-end reads at setting very-sensitive, end-to-end alignment. With these parameters, reads were only mapped against the reference (in this case the contigs) if they aligned over the entire length of the read with no mismatches, using 20 iterations to assign a read. The sam file output was then used as input for the cytoscapeviz.pl script [43] to create connections, contig length and contig coverage tab files [44]. The connections file was then used in Cytoscape [45] to create the networks, with size of the nodes relative to contig length and its color relative to contig coverage. Networks were given a putative taxonomic assignment if at least one of the contigs gave BLASTx results with the nr database at *e* values <0.001 or BLASTp against the RefSeq virus database including conserved domain search (*e* values <0.001), the latter performed by MetaVir. When multiple taxonomic assignments were found, the assignment was made at a higher taxon. For example, a contig with genes showing similarity to the *Podoviridae* family and another with genes similar to those of the family *Siphoviridae* within the same network resulted in an assignment of the order *Caudovirales* for the network.

Phylogenetic trees were generated with MEGA6 [46] using clustalW [47] at default settings for the alignment and the maximum likelihood (ML) method with 100 bootstraps for tree generation (Jones-Taylor-Thornton model for amino acids [48], Nearest-Neighbor-Interchange ML heuristic method).

For the read mapping analysis of GS and SS, the read files were merged into one file per metavirome. An additional phiX removal step was included as well as a removal of potential human contamination (hg18 bowtie2 reference index) using bowtie2 at setting very-fast, resulting in 15,207,796 reads for GS and 15,465,794 reads for SS. A reference set of metaviromes was downloaded from MetaVir and GenBank as fasta files and used to build a reference with bowtie2-build at the default settings. Alternatively, the GS and SS contigs were used as a reference. Mapping was performed at the very-fast setting using unpaired reads, which means that the mapping algorithm used only 5 iterations before assigning a mapped read which needed to align over its entire length with no mismatches allowed. Some metavirome files could not be converted into reference index files due to their large sizes. To assess the contigs to which the reads were mapped, the samtools package was used to extract all the mapped reads (samtools view—F 4) [49].

To cluster metaviromes based on the known virus fraction, the taxonomic composition of all dsDNA contig datasets available from MetaVir was downloaded (January 2015), including those of the Namib saline sites, GS contigs and SS contigs. The table was expanded to the species level, loaded into R and transformed into a presence/absence table. Bray-Curtis dissimilarities were calculated using the Vegan package, clustered using hclust, and subsequently plotted in R.

GS and SS contigs were annotated using the MetaVir pipeline, using MetaGeneAnnotator software [50] and BLASTx to the RefSeq virus database. In parallel, the same contigs were annotated by the VIROME pipeline using MetaGene [51] and BLASTp to the UNIREF100P [52] and MetaGenomes OnLine databases [41,53]. Annotated contigs of interest were downloaded from MetaVir as GenBank files. Additional annotations were found using the Conserved Domain search of NCBI [54]. The contig comparison figure of the salterproviruses was created using Easyfig [55] with tBLASTx for comparison and was annotated in Paint.net 4.0.5 (dotPDN LLC).

## 3. Results and Discussion

### 3.1. The Hosabes and Eisfeld Playas

The Hosabes spring, source of the Gobabeb Saline (GS) metavirome, had a variation in pH from 7.0–7.5 at the source and 9.0–9.5 at the sink, measured over multiple years (2010–2015). The electrical conductivity also displayed a range (61–103 mS/cm) with the lowest conductivities measured at the source and the highest at the sink. This translated into a total dissolved solids (TDS) range of approximately 39,000–66,000 ppm, giving a measure of the number of unbound inorganic solutes such as Na^2+^, Cl^−^, Mg^2+^, SO_4_^2−^, and CO_3_^2−^ present within the spring water. The salinity range based on the measured conductivity was 4.1%–7.5%. For the Eisfeld playa, where the Swakopmund Saline (SS) metavirome was sampled, the pH ranged from 6.5 at the source to 8.5 at the sink. The conductivities for this site reached a higher maximum, ranging from 66–180 mS/cm, giving a TDS range of 42,000–115,000 ppm and a salinity range of 4.5%–8.6%. These values agree well with those reported previously [31,33]. These salinity values are in the same range as the moderately hypersaline Salton Sea (CA, USA) which has a salinity of 5% in the water table and up the 11.8% in the sediment [56]. However, compared with solar salterns and crystallizer ponds which have reported salinity values of 13.8%–37%, these values are rather low, which can explain the novelty of these metaviromes [3,4,26,57].

### 3.2. Identification of Unknown Contigs and Reads Shows an Extended Range of ssDNA Viruses with a High Prevalence of Members of the Microviridae Family

The taxonomic breakdown of the Gobabeb (GS) and Swakopmund Saline (SS) metaviromes is described in Table 1 and is characterized by the high incidence of unknown reads (>92%) and contigs (>79%). At the gene level, 47% and 48% of the predicted proteins, for GS and SS, respectively, had no database homolog and were designated ORFans.

**Table 1:** Taxonomic breakdown of Gobabeb Saline and Swakopmund Saline metaviromes according to the MetaVir and VIROME pipelines.

Taxonomic Breakdown ^1^	Gobabeb Saline (GS)	Swakopmund Saline (SS)
Unknown reads (MetaVir)	95%	92%
Unknown contigs (MetaVir)	80%	79%
ORFans (VIROME)	47%	48%
Viral metagenomic ORFs (VIROME)	7%	9%
Microbial metagenomic ORFs (VIROME)	3%	5%
ORFs designated functional proteins (VIROME)	31%	30%
ORFs designated unassigned proteins (VIROME)	11%	8%

^1^ MetaVir uses BLASTp comparisons to the virus RefSeq database, while VIROME categorizes ORFs based on BLASTp comparisons with the UniRef100P database for functional and unassigned proteins and MGOLD for the remaining ORFs. “Unknown” refers to contigs and reads without equivalents in the searched database, ORFans are ORFs which have no homologous gene present in any of the databases.

**Figure 2:**
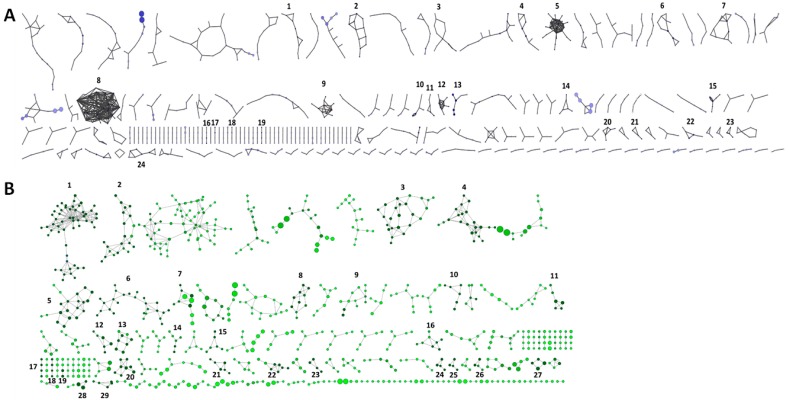
Network representation of the contigs of the Gobabeb Saline (**A**) and Swakopmund Saline (**B**) metaviromes. Connections between the contigs (circles) are based on read pairs mapping to different contigs. The circle size is proportional with contig length and the darker the color (light to dark blue for GS, light to dark green for SS), the higher the coverage of the reads mapping to a specific contig. Contig networks containing at least one contig with coverage higher than 1000× were investigated and are numbered. Only the contigs connected to at least one other are shown.

To provide a general taxonomic assignment to the unknown reads and contigs, we performed a network analysis. The networks in Figure 2 represent contigs of the metaviromes of Gobabeb (A) and Swakopmund (B) playas which connected to at least one other, where the size of the circle is proportional to contig size and the intensity of the color directly proportional to the coverage of the read mapping. The edges denote the read pairs which map to different contigs, thus connecting them in the network. For two contigs to be connected in a network, paired reads needed to be mapped to the different contigs over the full length of the read. In this case, an average of 172–174 bp (= average read length for these datasets) is then shared between the contigs at a 100% match. With this level of sequence similarity, we hypothesize that the contigs in a single network are taxonomically related. This analysis was adapted from Albertsen and colleagues (2013) where they used genome coverage and tetranucleotide binning of contigs followed by paired-end network scaffolding to generate single bacterial genomes from metagenomic datasets [44]. In this case, we have skipped the coverage and tetranucleotide binning to group both rare and abundant viral genomes which show a high level of sequence similarity over a section of the contigs. Additionally, tetranucleotide usage patterns for bacteriophages have been shown to be more consistent for the host range than within the same phage family [58], making this parameter inappropriate for viral binning for taxonomic assignment. Thus, the networks do not represent single genomes, but rather a set of genomes which can then be grouped at the (sub)family level or higher.

For the Gobabeb saline spring, 24 networks contained at least one contig with over 1000-fold coverage (Figure 2A, Table 2 and Table S1). Eight networks, comprising 36 contigs, were identified as ssDNA viruses, either not further characterized (networks 3, 5, 8, 16) or belonging to the families *Inoviridae* (network 2), *Microviridae* (networks 9, 10), or *Geminiviridae* (network 17). While the majority of contigs belonging to the families *Inoviridae* and *Microviridae* most likely represent bacteriophages, the *Geminiviridae* family contains plant viruses of which members of the genera *Mastrevirus* and *Begomovirus* have been known to infect tropical plants/crops in Sub-Saharan Africa [59]. Two of the ssDNA networks (5 and 16) showed identity to hypothetical proteins from sequences designated as an uncultured marine virus. This sequence is derived from a collection of environmental ssDNA viruses from ocean samples (British Columbia Strait of Georgia, Gulf of Mexico and Saanich Inlet) which are divided into 129 genetically distinct groups showing a high sequence divergence and no significant sequence similarity to known virus families [60]. The authors have suggested that these ssDNA genomes represent new virus families and we can hypothesize that many of the networks in this study also represent new lineages of ssDNA viruses. Four of the 24 networks showed similarity with proteins of bacteria, unicellular algae or fungi, suggesting a low level of contamination, transduced host DNA or the presence of integrated viruses in the reference database DNA. One network seemed to represent a temperate bacteriophage of the *Myoviridae* family while the remaining 11 networks were completely unaffiliated. For the 24 networks, 96 of the 108 contigs were classified as unknown environmental sample by MetaVir. As a result of the network assignments, we managed to increase the number of annotations and now only 39 out of 108 contigs remain completely unknown.

**Table 2:** Read mapping results of Gobabeb Saline contigs, resulting in the networks in Figure 2A. Putative taxonomic assignment is based on BLASTx results with the nr database at *e* values <0.001 and BLASTp against the RefSeq virus database including conserved domain search (*e* values <0.001).

Network ^1^	Number of Contigs ^2^	Maximum Contig Coverage	Average Network Coverage	Putative Taxonomic Assignment of Network	Marker Genes Present
1	5	1204×	739×	unknown	-
2	6	5193×	2849×	*Inoviridae*	Assembly protein
3	6	20,648×	4037×	ssDNA viruses	Rep protein, capsid protein
4	5	1247×	646×	unknown	-
5	12	77,280×	24,754×	ssDNA viruses	-
6	5	1083×	430×	unicellular algae	-
7	6	1070×	562×	unknown	-
8	15	90,340×	16,676×	ssDNA viruses	Coat protein, rep protein
9	6	159,565×	40,887×	*Microviridae*	protein D
10	3	4599×	1568×	*Gokushovirinae*	VP1, replication initiation protein
11	3	1660×	885×	bacteria	-
12	5	1733×	1128×	bacteria	-
13	3	1600×	898×	*Myoviridae*	Integrase, thioredo×in, primase
14	3	4477×	4096×	unknown	-
15	3	17,074×	6620×	unknown	Replication initiation factor
16	2	9629×	4971×	ssDNA viruses	-
17	2	73,466×	36,764×	*Geminiviridae*	Coat protein
18	2	72,629×	36,423×	unknown	-
19	2	28,306×	16,364×	unknown	-
20	3	3887×	2776×	unknown	-
21	2	8056×	4856×	unknown	-
22	3	1011×	522×	unknown	-
23	2	10,800×	8738×	unknown	-
24	4	1909×	992×	bacteria, fungi	-

^1^ 24 networks out of 163 were analyzed; ^2^ The 163 networks grouped 567 contigs out of 11,304, of which 2483 were positively identified as viral.

In the Swakopmund Saline dataset, 29 networks had at least one contig with over 1000-fold coverage, for a total of 154 contigs (Figure 2B, Table 3 and Table S1). The same trend as for the Gobabeb Saline metavirome was observed here, with 14 ssDNA virus networks with similarity to either the families *Circoviridae* (networks 8, 10, 11, 15, 21, 22), *Microviridae*, specifically the subfamily *Gokushovirinae* (networks 2, 6), *Inoviridae* (network 20), or to unassigned ssDNA viruses (networks 1, 4, 9, 24, 25). The increased presence of circoviruses, the majority of which infect avian species [59], in the Swakopmund saline site compared with the Gobabeb site could be explained by the site’s geographical location, closer to the sea and potentially frequented by a greater number of bird species [61]. Many of the contigs in the unidentified ssDNA networks showed similarity to one or more of the uncultured marine ssDNA viruses indicating that these are not exclusively marine viruses. One network was associated with tailed phages (network 7) and four with bacteria (networks 5, 12, 13, 28), of which one showed similarity to a replication protein of Halobacteria (network 13), possibly of viral origin. The remaining 10 networks were unknown. In this case, we were able to increase the number of affiliated contigs from 39–110 out of 154.

**Table 3:** Read mapping results of Swakopmund Saline contigs, resulting in the networks in Figure 2B. Putative taxonomic assignment is based on BLASTx results with the nr database at *e* values <0.001 and BLASTp against the RefSeq virus database including conserved domain search (*e* values <0.001).

Network ^1^	Number of Contigs ^2^	Maximum Contig Coverage	Average Network Coverage	Putative Taxonomic Assignment of Network	Marker Genes Present
1	27	445,272×	49,366×	ssDNA viruses	Rep protein
2	11	2553×	1327×	*Gokushovirinae*	Capsid protein, replication initiator, portal protein
3	12	2708×	1534×	unknown	Replication initiator domain
4	11	20,653×	6595×	ssDNA viruses	Rep protein, coat protein
5	10	7461×	3782×	bacteria	Rep protein
6	13	2449×	1261×	*Gokushovirinae*	Capsid protein, replication initiator
7	5	1640×	741×	*Caudovirales*	VirE, integrase
8	4	7756×	2660×	*Circoviridae*	Rep protein
9	6	1055×	412×	ssDNA viruses	Rep protein
10	3	1178×	992×	*Circoviridae*	Rep protein
11	3	5901×	3905×	*Circoviridae*	Rep protein
12	3	4081×	2463×	bacteria	-
13	4	1599×	945×	Halobacteria	Rep protein
14	3	1645×	838×	unknown	-
15	3	2242×	1207×	*Circoviridae*	Capsid protein
16	4	1480×	812×	unknown	-
17	2	1287×	1123×	unknown	-
18	2	2230×	1638×	unknown	-
19	2	1118×	600×	unknown	-
20	4	9613×	6416×	*Inoviridae*	Assembly protein
21	3	1578×	1178×	*Circoviridae*	Capsid protein
22	4	6853×	3898×	*Circoviridae*	Replication-associated protein
23	2	79,472×	49,830×	unknown	-
24	2	9622×	7527×	ssDNA viruses	Replication-associated protein
25	2	17,505×	10,399×	ssDNA viruses	Capsid protein
26	2	1782×	1208×	unknown	-
27	2	5295×	4949×	unknown	-
28	2	3670×	2825×	Cyanobacteria	-
29	3	2157×	1447×	unknown	-

^1^ 29 networks out of 98 were analyzed; ^2^ The 98 networks grouped 397 contigs out of 22,352, of which 4886 were positively identified as viral.

In both GS and SS datasets, several contigs with phage integrase genes were identified (see Table 2 and Table 3). In the GS metavirome, 2483 contigs were recognized as viral with counterparts in the database of which 67 contained an integrase gene. For the 4886 viral contigs of SS, 92 contained an integrase gene. Taking into account that we have collected only extracellular viral particles, and have not used a lysogenic induction step [62], both metaviromes have a significant potential for lysogeny.

The high prevalence of ssDNA genomes in these datasets is most likely caused by the multiple displacement DNA amplification method (MDA), using the phi29 polymerase. Recent research has indicated that MDA preferentially amplifies circular ssDNA viruses when present [63,64,65].

**Figure 3:**
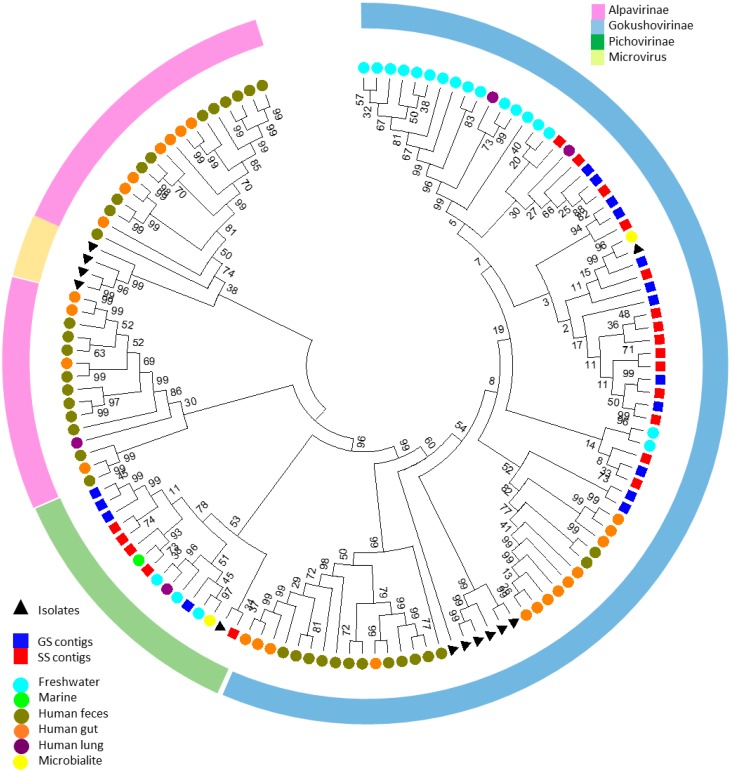
Maximum likelihood amino acid phylogenetic tree of the predicted capsid proteins (VP1) of presumed members of the *Microviridae* family. Sequences from this study are indicated by squares, known isolates by triangles and capsid proteins extracted from assembled genomes from previous metagenomic studies by circles [66]. The arches represent the presumed taxonomic distribution of the genomes. The tree with the highest log likelihood (−104,932.0787) is shown. Initial tree(s) for the heuristic search were obtained by applying the Neighbor-Joining method to a matrix of pairwise distances estimated using a Jones-Taylor-Thornton model. The analysis involved 133 amino acid sequences. There were a total of 1145 positions in the final dataset. Bootstrap percentages (on 100) are indicated at the branches.

In the network analysis, many contigs with similarity to marine gokushoviruses, or *Microviridae* members in general, were identified. To investigate the presence and diversity of potential genomes belonging to this family, all presumed circular contigs with lengths over 4000 bp were extracted from the datasets through MetaVir, and the major capsid protein amino acid sequences were compared to those of selected isolates of the family *Microviridae* and the major capsid proteins (VP1) of a set of 81 assembled genomes from metavirome datasets [66]. The resulting phylogenetic tree (Figure 3) placed the saline contigs within clades of the subfamilies *Gokushovirinae* and “Pichovirinae”. The gokushovirus group was the most diverse, containing the majority of the genomes of the Namib saline metaviromes, as well as genomes of marine, freshwater and human virome origins. With these additional capsid protein sequences, there seems to be a mostly environmental clade and two human-associated clades within the subfamily *Gokushovirinae*, with two GS capsid proteins clustering with a human-associated clade. It is possible that the two GS genes belong to phage genomes associated with bacteria present in animal feces (observed in and around the salt pans). A division into two groups was also witnessed after a PCR amplification study, giving rise to a eukaryote-associated clade and an environmentally-dominated clade [67]. Additionally, our results confirm that the *Gokushovirinae* subfamily is ubiquitously distributed and therefore probably of importance in general microbial ecology [67,68,69]. A number of saline contigs of both sites clustered with the “Pichovirinae” clade, which is an infrequently recovered proposed subfamily, comprised of sequences of aquatic origin, including freshwater, coral and microbialite metaviromes [66]. Additional studies have identified three new pichovirus-related sequences, one from deep sea trench sediment and two from methane seep sediments [67,70]. However, the diversity of pichoviruses might be underestimated at this time, since the PCR and RFLP-based studies seem to favor amplification of gokushoviruses related to the *Chlamydia* phage isolates which were used for primer design [67].

No capsid proteins of the saline metaviromes in this study were clustered with the “Alpavirinae” or members of the genus *Microvirus*. Alpaviruses have so far only been found in human-associated viromes [66,71]**,** and the lack of sequences clustering with this group could indicate an absence of human contamination. It is interesting to note that in this analysis, the phiX174-related viruses of the *Microvirus* genus form a clade which is nested within the “Alpavirinae” clade, albeit with low bootstrap support. Two distinct clades of alpaviruses are also found in the phylogenetic analysis of Roux and colleagues, indicating that this subfamily could be split as more microviruses are sequenced [66].

### 3.3. The Gobabeb Saline Site Contains Novel Haloviral Genomes Related to the Genus Salterprovirus

**Figure 4:**
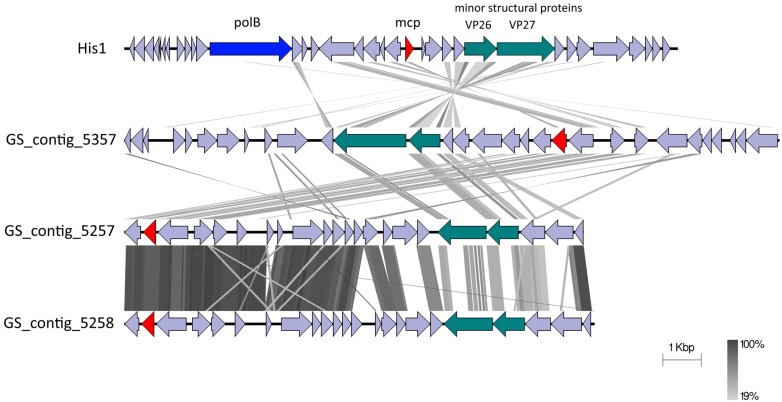
Pairwise genomic comparison of the salterprovirus His1 with metaviromic contigs from the Gobabeb Saline metavirome. The predicted coding sequences are depicted by arrows. The putative polymerase B gene is colored blue, the major capsid protein (mcp) red and the minor structural proteins in blue-green. Grey areas between the genomes are pairwise tBLASTx similarities (areas of amino acid sequence identity).

The saline metavirome datasets were mined for contigs showing resemblance to known haloarchaeal viruses. This analysis revealed 152 contigs for GS and 35 contigs for SS which showed varying degrees of similarity. The majority of these signatures belonged to viruses informally categorized as Haloviruses, comprising a group of head-tailed archaeal viruses with myovirus or siphovirus morphologies [21]. To investigate potential new lineages of haloarchaeal viruses, we examined contigs with a length approximating that of the genome to which they showed the most similarity. Three large contigs linked to salterprovirus His1 (the only representative of its genus) were identified in the Gobabeb saline dataset for which the read mapping showed a coverage from 15×–60× (depicted in Figure 4). His1 is a spindle-shaped haloarchaeal virus infecting the euryarchaeyote *Haloarcula hispanica* and has been isolated from Lake Victoria, Australia [72,73]. GS contigs 5257 and 5258 showed the highest similarity at the amino acid level, although average nucleotide identities could not be calculated due to very low reciprocal similarity values. The three GS contigs showed amino acid sequence similarities to several genes of His1. Three structural protein-encoding genes of His1, the major capsid protein (mcp) and two minor structural proteins (VP26 and VP27) [74], had counterparts in the metavirome contigs be it at low amino acid similarities. The putative DNA polymerase, PolB, however, could not be identified in the metavirome contigs. One reason could be that the contigs do not represent complete genomes, but it is also possible that these viruses do not encode their own DNA polymerases. The C terminal region of the minor structural protein VP27 of His1 seemed to be conserved in the metavirome contigs and the proteins of both His1 and contig 5357 contained a carbopeptidase regulatory-like domain. With this low level of amino acid similarity and no significant nucleic acid identity, the GS contigs do not represent genomes belonging to the genus *Salterprovirus*. Rather, taking into account the conservation of the structural proteins, these genomes could be the first representatives of a candidate “Salterproviridae” family.

**Figure 5:**
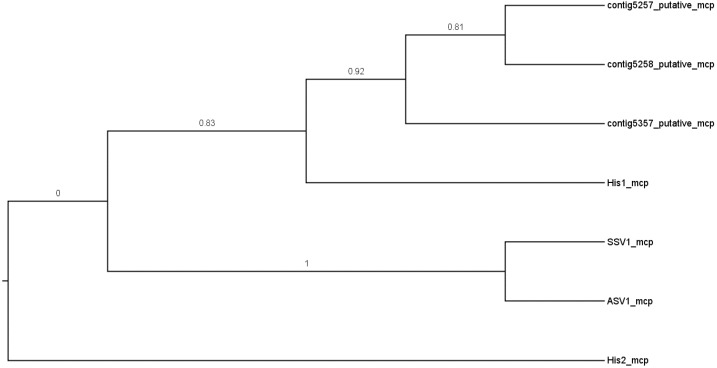
Maximum Likelihood phylogenetic tree of the predicted major capsid proteins (mcp) of the GS metavirome contigs, the salterprovirus His1, the proposed gammapleiolipovirus His2 and representative isolates of the family *Fuselloviridae*, *Sulfolobus* spindle-shaped virus 1 (SSV1) and *Acidianus* spindle-shaped virus 1 (ASV1). The tree with the highest log likelihood (−1555) is depicted here, showing only the topology. Bootstrap values (calculated on 100) are shown for each branch. The final alignment contained 142 amino acid positions.

As the capsid gene is widely used as a phylogenetic marker, the evolutionary relationship between VP1 of the three putative genomes and isolated archaeal viruses was investigated in an amino acid-based phylogenetic tree (Figure 5). We included the fuselloviruses *Sulfolobus* spindle-shaped virus 1 (SSV1) and *Acidianus* spindle-shaped virus 1 (ASV1) which share morphological characteristics with His1, and the newly classified gammapleolipovirus His2 (ICTV Proposal 2015.042a-oB, ictvonline.org) which infects the same host as His1. The three metaviromic putative capsid proteins clustered with that of His1 virus, and were also distantly related to the fuselloviruses, while His2 was practically unrelated. All putative capsid proteins contained two hydrophobic transmembrane domains (data not shown) which were found to be characteristic for fusello-like archaeal viruses (or tailless spindle-shaped viruses) but are not present in members of the family *Bicaudaviridae* (tailed spindle-shaped viruses) [74,75]. Based on this information, we can hypothesize that the candidate family “Salterproviridae”, comprising the genus *Salterprovirus* and the metavirome contigs, shares an evolutionary relationship with fuselloviruses. Alternatively, as previously suggested by Krupovic and colleagues, all tailless spindle-shaped viruses could be grouped into the family *Fuselloviridae* and be recognized as different genera, with the genus *Salterprovirus* becoming “Epsilonfusellovirus” [75].

### 3.4. The Gobabeb and Swakopmund Saline Metaviromes are Novel and More Closely Related to Each Other Than to Other Metaviromes

#### 3.4.1. Read Mapping

To investigate the degree of similarity between GS, SS and other metaviromes, two strategies of read mapping were used. In a first strategy, reads from a set of 44 publically available metaviromes, including ssDNA only and hypersaline metaviromes, were mapped to the GS or SS contigs (Table S2). In the second strategy, the reads from GS or SS were mapped against the reads or contigs from the environmental metaviromes.

Of the Gobabeb Saline reads, 92.7% mapped to their own contig dataset, illustrating that the assembly represented almost the entire read dataset. For the Swakopmund data, this was slightly higher at 93.2%. When mapping the two metaviromes against each other, 57.7% of the GS reads mapped to the SS contigs and 55.4% of the SS reads mapped to the GS contigs. This very high degree of similarity between the two saline metaviromes is indicative of a high similarity in the viral community structures, supporting the hypothesis that these similar environments (shallow, desert salt springs) select for a similar community composition despite the geographic distance (approximately 124 km apart).

Since MDA preferentially amplifies ssDNA, we compared the saline reads and contigs with three ssDNA metavirome datasets collected from different ocean regions [60]. This yielded no overlap with the reads from the British Columbia Strait of Georgia (SOG) and a low overlap for the Saanich Inlet and the Gulf of Mexico ssDNA reads (Table S2), indicating that the saline metavirome ssDNA complement is very distinct from the marine.

Comparing the GS and SS reads and contigs with other saline metaviromes, we found that the Namib sites were markedly novel. For the solar saltern metaviromes (MetaVir project IDs 24-32; [1]), no similarity was found with the Namib sites (max. 20 reads mapped) while the hypersaline metaviromes from the unpublished “Archevir” project (P-series, MetaVir project IDs 1700-1705) gave a maximum overlap of 131 reads.

For the Gobabeb saline metavirome, over 1000 reads mapped to the reads of freshwater Lake Michigan metaviromes (MetaVir project IDs 3305, 3306), and to a seawater metavirome read dataset, Indian Ocean virome sample GS108 (MetaVir project ID 1478, [76]). The disparity between the viromes mapped to reads and contigs could be explained by the presence of identical reads in the datasets. The presence of similar reads in these very different datasets seemingly indicates the presence of a small fraction of ubiquitous viral genomes. For the SS dataset, less similarity was found, with only the Indian Ocean GS108 sample showing >1000 mapped reads.

The results of the read mapping of GS and SS reads against metavirome contigs revealed a high degree of similarity between both GS and SS, and the Antarctic hypolith (AH) and open soil (AOS) metaviromes (MetaVir project IDs 2472, 2473; [77]). Given the geographical spread of these metavirome origins and the difference in habitat, we analyzed the contigs to which the reads were mapped. In the case of GS, all the reads mapped to four AH contigs with the majority of the reads (275,746) mapped to a contig showing resemblance to a hypothetical protein of *Cellulophaga* phage phi47:1, a myovirus isolated from seawater [78]. This exact same protein was responsible for the majority of the mapped reads in the AOS contig (95,629). Phage phi47:1 has been described as belonging to a highly divergent group of myoviruses (CbaSM-like phages) with ~54 kb genomes present in ocean environments. This result indicates the potential globally-distributed nature of a related phage group [78]. This was in contrast with the SS read mapping to the same metaviromes, in which a *Circoviridae* capsid protein had the majority of mapped SS reads (40,836). For the AOS contigs, the SS reads mapped to a range of contigs described as environmental samples in MetaVir, with some hits to the virus families *Myoviridae*, *Mimiviridae* and *Nudiviridae*. We therefore conclude that the seemingly high similarity between the Antarctic soil and the Namib saline metaviromes is caused by a limited number of shared genes possibly belonging to a small fraction of globally ubiquitous viruses.

#### 3.4.2. Comparison of the Presence/Absence of Viral Groups

**Figure 6:**
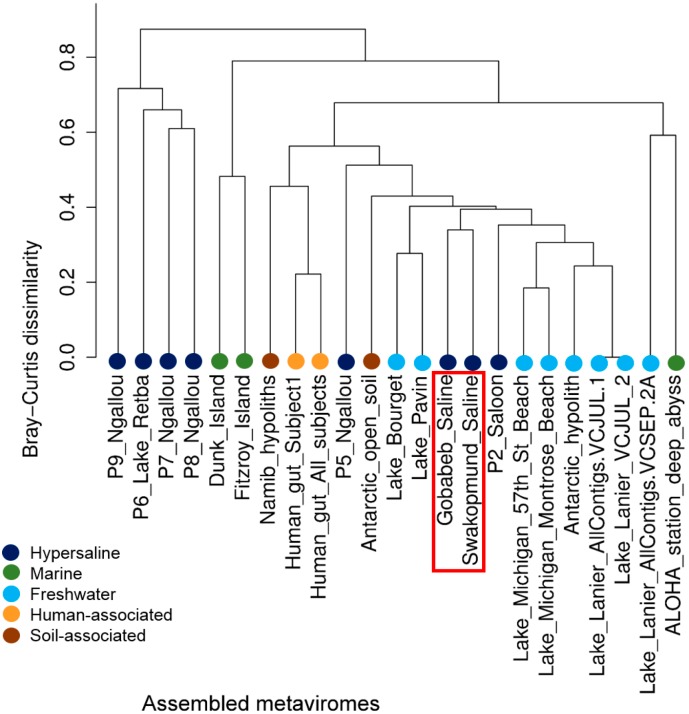
Cluster analysis of a Bray Curtis dissimilarity matrix based on the presence/absence of viral species in the investigated metaviromes as found by the MetaVir pipeline. Metaviromes were colored according to their biome. The Gobabeb Saline and Swakopmund Saline metaviromes are indicated with a red box.

Since MDA of the metaviromic DNA is likely to introduce a bias towards ssDNA virus taxa, we conducted the comparison of the known viral groups solely based on presence/absence of viral species in the contig datasets. Hierarchical clustering of the Bray-Curtis dissimilarity matrix confirmed the result from the read mapping data, that the Gobabeb and Swakopmund saline metaviromes were most closely related to each other (Figure 6). All the viromes used in this comparison showed at least some shared species as the dissimilarity never reached 1. This implies a baseline of viral species shared over a wide range of environments. The GS and SS metaviromes were part of a very heterogeneous cluster comprising mostly freshwater lake environments but also hypoliths and human gut viromes. The presence of such a heterogeneous cluster is likely due to a database bias, seeing as this analysis only covers the known fraction of viruses, and/or the use of a small reference virome set. The seawater viromes from the Great Barrier Reef (Dunk and Fitzroy Island) and the hypersaline Archevir project (P-series, MetaVir project IDs 1700-1705) clustered separately. No virus taxa were unique to the Namib saline metaviromes. As expected from the library construction process, no RNA viruses were observed, and neither were the Archaea-infecting families *Fuselloviridae* and *Rudiviridae* which specifically infect extremely thermophilic and/or acidophilic hosts.

We also noted the absence of the recently discovered crAssphage, a genome which was cross-assembled from human gut metaviromes and which has been shown to be ubiquitously distributed in public metagenomes [79]. Shortly after its discovery, crAssphage was already suggested as a marker to track human fecal pollution [80], from which we can infer an absence of human fecal contamination in the Namib saline sites.

## 4. Conclusions

The Namib saline metaviromes share a high degree of similarity in their sequence space and are distinct from other published metaviromes. MDA of the virome DNA has revealed a high richness of ssDNA viruses, from potential phages to insect, plant and bird virus signatures. Almost all of the known haloarchaeal virus genomes have homologs in these metaviromes, suggesting that these salt pans are ideally suited for the discovery of new archaea-infecting viruses. Furthermore, the presence of many tailed phages makes these sites useful targets to mine for phage enzymes such as DNA polymerases for industrial applications.

## Supplementary Materials

The following are available online at http://www.mdpi.com/1999-4915/8/1/14/s1, Table S1: Network analysis annotation results (a) Gobabeb Saline metavirome, (b) Swakopmund Saline metavirome, Table S2: Read mapping results for the Namib Saline metaviromes (Gobabeb and Swakopmund Saline) to other publically available metaviromes.
