# Supplementary material for: Metaviromics of Namib Desert Salt Pans: A Novel Lineage of Haloarchaeal Salterproviruses and a Rich Source of ssDNA Viruses
Source: Viruses. 2016 Jan 8;8(1):14. doi: 10.3390/v8010014 (PMC4728574; doi:10.3390/v8010014)
Supplement: Supplementary file 1 [file viruses-08-00014-s001.zip › viruses-105643-supplementary/viruses-105643-S2.pdf]

Supplementary Materials: Metaviromics of Namib Desert Salt Pans: A Novel Lineage of Haloarchaeal Salterproviruses and a Rich Source of ssDNA Viruses

Evelien M Adriaenssens [1,*], Leonardo Joaquim van Zyl [2], Don A Cowan [1] and Marla I Trindade [2]

Table S2. Read mapping results for the Namib Saline metaviromes (Gobabeb and Swakopmund Saline) to other publically available metaviromes.

Metavirome	Biome	Number of Reads	Sequencing Platform	Reference	Metavirome Reads Mapped against GS Contigs	GS Reads Mapped against Metaviromes	Metavirome Reads Mapped against SS Contigs	SS Reads Mapped against Metaviromes
GS reads	Saline	15,207,796	Illumina	This study	14,098,128	ND	8,767,600	ND
SS reads	Saline	15,833,257	Illumina	This study	8,220,907	ND	14,749,408	ND
GOM ssDNA	Seawater	87,274	454	[43]	0	179	2	111
Saanich Inlet ssDNA	Seawater	96,950	454	[43]	0	3848	0	ND
SOG ssDNA	Seawater	95,402	454	[43]	0	33	0	ND
Antarctic Lake Spring	Fresh water	41,322	454	[81]	0	23	0	0
Antarctic Lake Summer	Fresh water	38,475	454	[81]	1	328	0	0
Lake Pavin	Freshwater	649,290	454	[49]	2	ND	4	ND
Lake Bourget	Freshwater	593,084	454	[49]	29	ND	36	ND
Hamdoun	Freshwater	39,404	454	[82]	0	11	1	1
El Berbera	Freshwater	75,921	454	[82]	8	34	5	5
Ilij	Freshwater	62,059	454	[82]	2	30	1	1
Molomhar	Freshwater	75,709	454	[82]	3	20	0	0
FTR July 2007	Freshwater	116,107	454	[83]	0	4	0	0
FTR Aug 2007	Freshwater	96,373	454	[83]	3	263	1	1
FTR Jan 2008	Freshwater	76,396	454	[83]	0	11	0	0
Lake Michigan 57th Street Beach	Freshwater	296,610	Illumina	MetaVir Project ID 3305	209	14,684	18	338
Lake Michigan Montrose Beach	Freshwater	295,664	Illumina	MetaVir Project ID 3306	463	30,349	34	358
Saltern Low 0704	Saline	268,049	454	[1]	9	20	11	11
Saltern Low 111005	Saline	109,836	454	[1]	1	9	1	1

Saltern Low 112805	Saline	62,363	454	[1]	39	6	43	43
Saltern Medium 111005	Saline	39,348	454	[1]	1	0	0	0
Saltern Medium 112205	Saline	55,142	454	[1]	1	2	0	0
Saltern Medium 111605	Saline	58,319	454	[1]	3	2	2	2
Saltern High 111605	Saline	151,180	454	[1]	29	11	1	1
Saltern High 120705	Saline	46,628	454	[1]	7	5	4	4
Saltern High 112805	Saline	4536	454	[1]	11	10	7	7
Octopus hot spring	Hot spring	22,272	Sanger	[37]	1	360	0	0
Bearpaw hot spring	Hot spring	8352	Sanger	[37]	3	486	2	2
Salton Sea 1 Vir 20060823	Hypersaline	50,935	454	MetaVir Project ID 47	0	0	3	3
Salton Sea 2 Vir 20060823	Seawater	27,689	454	MetaVir Project ID 50	0	0	1	1
POV LA26S	Seawater	165,256	454	[84]	15	806	0	22
Indian Ocean GS108	Seawater	320,104	454	[59]	132	3659	44	2,240
Human gut subject 1 (contigs)	Host associated	3638	Illumina	[85]	ND	17	ND	12
Human gut all subjects (contigs)	Host-associated	10,202	Illumina	[85]	ND	334	ND	2,450
Antarctic Hypolith (contigs)	Soil-associated	32,574	Illumina	[60]	ND	428,098	ND	42,598
Antarctic Open Soil (contigs)	Soil	8442	Illumina	[60]	ND	153,397	ND	13,520
Namib Hypolith (contigs)	Soil-associated	4574	Illumina	[29]	ND	263	ND	17
Lake Lanier VCJUL1 (contigs)	Freshwater	23,132	Illumina	MetaVir Project ID 2734	ND	73	ND	5
Lake Lanier VCSEP2A (contigs)	Freshwater	1767	Illumina	MetaVir Project ID 2736	ND	0	ND	0
Dunk Island (contigs)	Seawater	3210	454	[84]	ND	6	ND	76
Fitzroy Island (contigs)	Seawater	2382	454	[84]	ND	22	ND	106
P2 Saloon (contigs)	Saline	7302	Illumina	MetaVir Project ID 1701	ND	112	ND	127
P5 Ngallou (contigs)	Saline	3018	Illumina	MetaVir Project ID 1700	ND	131	ND	98
P6 Lake Retba (contigs)	Saline	884	Illumina	MetaVir Project ID 1702	ND	53	ND	0
P7 Ngallou (contigs)	Saline	1312	Illumina	MetaVir Project ID 1703	ND	17	ND	10

ND: not determined due to large reference index sizes.

References

81. López-Bueno, A.; Tamames, J.; Velázquez, D.; Moya, A.; Quesada, A.; Alcamí, A. High diversity of the viral community from an Antarctic lake. *Science* **2009**, *326*, 858–861.
82. Fancello, L.; Trape, S.; Robert, C.; Boyer, M.; Popgeorgiev, N.; Raoult, D.; Desnues, C. Viruses in the desert: A metagenomic survey of viral communities in four perennial ponds of the Mauritanian Sahara. *ISME J*. **2013**, *7*, 359–369.
83. Tseng, C.-H.; Chiang, P.-W.; Shiah, F.-K.; Chen, Y.-L.; Liou, J.-R.; Hsu, T.-C.; Maheswararajah, S.; Saeed, I.; Halgamuge, S.; Tang, S.-L. Microbial and viral metagenomes of a subtropical freshwater reservoir subject to climatic disturbances. *ISME J*. **2013**, *7*, 2374–2386.
84. Hurwitz, B.L.; Sullivan, M.B. The Pacific Ocean Virome (POV): A marine viral metagenomic dataset and associated protein clusters for quantitative viral ecology. *PLoS ONE* **2013**, *8*, e57355.
85. Minot, S.; Wu, G.D.; Lewis, J.D.; Bushman, F.D. Conservation of gene cassettes among diverse viruses of the human gut. *PLoS ONE* **2012**, *7*, e42342.

© 2016 by the authors; licensee MDPI, Basel, Switzerland. This article is an open access article distributed under the terms and conditions of the Creative Commons by Attribution (CC-BY) license (http://creativecommons.org/licenses/by/4.0/).
